# Preparation of Nanostructured Sn/Ti Oxide Hybrid Films with Terpineol/PEG-Based Nanofluids: Perovskite Solar Cell Applications

**DOI:** 10.3390/ma16083136

**Published:** 2023-04-16

**Authors:** Saeid Vafaei, Vamsi Krishna Boddu, Stephen Jala, Pavan Kumar Bezawada, Nagisa Hattori, Seiho Higashi, Takashi Sugiura, Kazuhiro Manseki

**Affiliations:** 1 Mechanical Engineering Department, Bradley University, 1501 West Bradley Avenue, Peoria, IL 61625, USA; 2 Industrial and Manufacturing Engineering and Technology Department, Bradley University, 1501 West Bradley Avenue, Peoria, IL 61625, USA; 3 Graduate School of Natural Science and Technology, Gifu University, Yanagido 1-1, Gifu 501-1193, Japan

**Keywords:** sintering, TiO_2_, SnO_2_, nanoparticles, perovskite solar cells

## Abstract

Tin oxide (SnO_2_) and titanium dioxide (TiO_2_) are recognized as attractive energy materials applicable for lead halide perovskite solar cells (PSCs). Sintering is one of the effective strategies for improving the carrier transport of semiconductor nanomaterials. Using the alternative metal-oxide-based ETL, nanoparticles are often used in a way that they are dispersed in a precursor liquid prior to their thin-film deposition. Currently, the creation of PSCs using nanostructured Sn/Ti oxide thin-film ETL is one of the topical issues for the development of high-efficiency PSCs. Here, we demonstrate the preparation of terpineol/PEG-based fluid containing both tin and titanium compounds that can be utilized for the formation of a hybrid Sn/Ti oxide ETL on a conductive substrate (F-doped SnO_2_ glass substrate: FTO). We also pay attention to the structural analysis of the Sn/Ti metal oxide formation at the nanoscale using a high-resolution transmission electron microscope (HR-TEM). The variation of the nanofluid composition, i.e., the concentration of tin and titanium sources, was examined to obtain a uniform transparent thin film by spin-coating and sintering processes. The maximum power conversion efficiency was obtained for the concentration condition of [SnCl_2_·2H_2_O]/[titanium tetraisopropoxide (TTIP)] = 25:75 in the terpineol/PEG-based precursor solution. Our method for preparing the ETL nanomaterials provides useful guidance for the creation of high-performance PSCs using the sintering method.

## 1. Introduction

Thin solid films consisting of semiconductor nanomaterials have received increasing attention for their potential applications, such as optoelectronic devices [1,2,3]. Among them, perovskite solar cells (PSCs) built from multi-semiconductor layers including halide perovskite light absorbers are now attracting widespread interest, in view of not only the intriguing basic science of related materials but also their commercialization [4,5,6]. These devices are also expected to be integrated into versatile renewable energy systems [7]. In particular, with the need for alternative energy sources, materials research for PSC applications has gained tremendous progress in the improvement of the light-to-electricity conversion of PSCs, as well as the durability and diversity of devices with various transparent conductive substrates. One of the major device structures of PSCs realizing a decent power conversion efficiency includes the use of metal oxide nanoparticle layers that are deposited on top of conductive F-doped tin oxide (FTO) glass substrates [4]. These are known as mesoscopic-type or planar-type devices, for which lead halide perovskite is crystallized adjacent to the metal oxide layers. As for the bottom layer, the following two processes are alternatively employed: annealing-free deposition at temperatures as low as 100 °C toward the development of flexible PSCs and high-temperature sintering, generally treated at 450~500 °C using an electric oven. For the latter case, heating of the metal oxide precursor films can induce the fusion of the corresponding nanoparticles to ensure efficient electron transport in the device.

There have been several reports on the advantages of using multi-layer-type SnO_2_/TiO_2_ or Sn-doped TiO_2_ as an electron transport layer (ETL) in PSCs. As an example, Guo et al. found that TiO_2_/SnO_2_ nanocomposites prepared by a simple one-step process had high electrical conductivity and favorable energy band alignment at the interface of ETLs/lead halide perovskite layers [8]. They demonstrated that the inclusion of SnO_2_ nanoparticles in a compact TiO_2_ layer had a positive result in increasing power conversion efficiency (PCE). SnO_2_ nanoparticle powder was added into a TiO_2_ precursor solution to produce nanocomposites, followed by spin-coating and sintering at 500 °C. A maximum PCE of 16.8% was reported for CH_3_NH_3_PbI_3_-based solar cells, showing a superior PCE as opposed to that of TiO_2_-based PSCs.

Another strategy regarding a bilayer ETL was reported by Hu et al. [9]. They pointed out that cascading energy levels of integrated SnO_2_/TiO_2_ layers and TiO_2_-mediated defect passivation of SnO_2_ contributed to photo-excited energy loss. Here, the SnO_2_/TiO_2_ designed for the ETL was made of nanoparticles, in which colloidal SnO_2_ nanoparticles are commercial products based on solution processes. The deposition was carried out via subsequent spin-coating and annealing at 150 °C. As a result, a marked maximum light-to-electricity conversion efficiency of 20.50% was achieved when a multi-cation-type perovskite (FAPbI_3_)_1−x_(MAPbBr_3_)_x_ was deposited on top of the ETL. It was found that the bilayer structure led to a significant improvement in PCE when it was compared to that of the SnO_2_-only control device (18.09%). Additionally, the TiO_2_ overcoat for the SnO_2_ layer made it possible to enhance the long-term stability of the device.

In regard to the passivation of ETLs, Abuhelaiqa et al. [10] pointed out that SnO_2_ films in bilayer SnO_2_/TiO_2_ ETLs are effective in the improvement of the device stability of PSCs. The degradation of perovskite film, especially the interface between the ETL and perovskite, was retarded, and PbI_2_ formation could also be suppressed. The additional SnO_2_ was shown to reduce charge recombination at the material interface, and improved optical durability was also reported [10].

A low-temperature process has also been examined to investigate the effect of Sn doping in TiO_2_ on perovskite solar cell performance [11]. The authors demonstrated that Sn(IV) can be introduced into the TiO_2_ lattice, which was possible by the addition of SnCl_4_·5H_2_O into an aqueous TiCl_4_ solution. Film preparation was carried out at a temperature of less than 100 °C. As opposed to the performance of pristine TiO_2_, they showed more efficient electron extraction and transport, giving rise to a higher short-circuit current density (J_sc_). Their optimized solar cell employing Sn-doped TiO_2_ had a PCE of 17.2%. This was a 29.3% higher value than that of the non-doped TiO_2_ device. A higher charge carrier mobility of Sn-doped TiO_2_ compared to that of the non-doped type has also been reported by other authors [12]. They showed an optimized PCE of 15.4% by introducing 1.0 mol% Sn-doped TiO_2_ in their perovskite solar cell.

An ETL consisting of Sn-TiO_2_ was also processed on an FTO substrate by co-electrodeposition and changing the doping amount of Sn [13]. The authors mentioned that the deposition rate was faster than that of the pristine type because of less ohmic overpotential. The use of a 5% Sn-doped SnO_2_ ETL enabled the maximum PCE of 16.8% to be obtained, which was a 9.8% increase compared to that of the non-doped TiO_2_ device. They pointed out that improved carrier concentration, as well as the increased extraction capability for Sn doping, led to higher PCE.

Multi-component-type ETLs thus have significant potential to create high-performance PSCs. Further investigation into the relationships between Sn/Ti oxide 3D nanostructures and device performance would be a major issue to be addressed. The purpose of this research is focused on fully understanding the formation of micro-structured Sn/Ti oxide thin films. XRD and TEM measurements were carried out for the detailed analysis of Sn/Ti metal oxide samples, which were prepared by mixing Tin(II) chloride and Titanium(IV) isopropoxide in a suitable base liquid and additive. Notably, we found out that nanofluid preparation using a terpineol/polyethylene glycol (PEG) medium is a key process to obtaining a uniform thin film. In addition, the formation mechanism of Sn/Ti oxide nanoparticles involving the elimination of the base liquid and additive was clarified. The sintering effects of nanostructured semiconductor layers on device performance were discussed towards the development of lead halide PSCs showing high efficiencies.

## 2. Materials and Methods

### 2.1. Chemicals

Titanium tetraisoproxide (TTIP, >97.0%), N,N-dimethylformamide (DMF, dehydrated, >99.5%), dimethyl sulfoxide (DMSO, dehydrated, 99.0%), 2-Propanol (IPA, dehydrated, >99.7%), and acetonitrile (dehydrated, >99.5%) were purchased from Kanto Chemical Co., Inc. (Portland, OR, USA). Spiro-MeOTAD, chlorobenzene (CB, anhydrous, 99.8%), Terpineol (anhydrous), and 4-tert-butylpyridine (TBP, 98%) were obtained from Sigma-Aldrich, Co. (St, Louis, MI, USA). Methylamine hydrobromide (MABr, >98.0%), Cesium iodide (CsI, >99.0%), Formamidine hydroiodide (FAI, 99.99%), Lead(II) iodide (PbI2, 99.99%), Lithium bis(trifluoromethanesulfonly)imide (Li-TFSI, >98.0%), and Formamidine hydrobromide (FABr, 99.99%) were purchased from Tokyo Chemical Industry Co., Ltd. (Tokyo, Japan). Polyethylene glycol 400 (PEG400) and Tin(II) chloride dihydrate (SnCl_2_·2H_2_O, >97.0%) were from FUJIFILM Wako Pure Chemical Corporation, Richmond, VA, USA. All chemicals were used as purchased.

### 2.2. Preparation of Sn/Ti Oxide Thin Films

F-doped tin oxide glass substrates (11 Ω/□ Nippon Sheet Glass, Tokyo, Japan) were washed and cleaned with UV–ozone treatment. We first mixed 40 wt% (0.7 g) of ethanol with 40 wt% (0.7 g) of terpineol as a base liquid and then added 20 wt% (0.35 g) of PEG400 to prepare a base solvent.

Several precursor solutions changing the concentrations of metal sources were prepared as follows: To the mixture of base liquid and PEG, SnCl_2_·2H_2_O and TTIP were added. The ratios of the metal sources were [SnCl_2_]/[TTIP] = 100:0, 75:25, 50:50, 25:75, 0:100 (mol/mol). The concentrations of the metal sources in all those solutions were 10 wt%. For example, 0.39 g of SnCl_2_·2H_2_O was used for preparing the 1:0 condition. Typically, the 10 wt% solution containing metal sources was stirred for 30 min at ambient temperature, followed by spin-coating on top of the FTO substrate at 3000 rpm for 30 s. The substrates were immediately heated on a hot plate at 120 °C for 20 min.

### 2.3. Sintering Process

**Figure 1:**
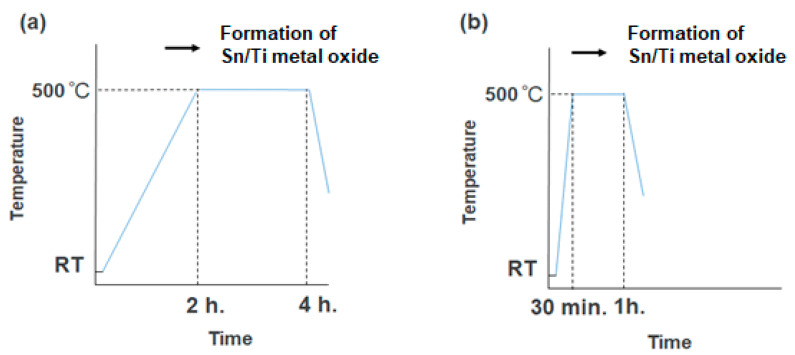
Sintering strategies of the Sn/Ti oxide thin-film samples in air. The periods of maximum temperature at 500 °C were 2 h for (**a**) and 30 min for (**b**), respectively. From 500 °C, the samples were cooled down to room temperature naturally.

The substrates were placed in an electric furnace, KDF 300 Plus (DENKEN-HIGHDENTAL, Kyoto, Japan) for the sintering process. In regard to the sintering strategy, the temperature was increased from room temperature to 500 °C. The two different ramping times of 2 h and 30 min were examined. After the maximum temperature was kept at 500 °C, it was then cooled down naturally. The periods of maximum temperature were 2 h and 30 min, respectively (see Figure 1).

### 2.4. Evaluation of Sn/Ti Oxide Thin Films

Nanostructures of TiO_2_ films after nanofluid pool boiling were characterized by scanning electron microscopy (SEM) of HITACHI S-4800 (Hitachi, Ltd., Tokyo, Japan). The transmittance spectrum of the Sn/Ti oxide film was measured using PerkinElmer Lambda950 (PerkinElmer, Waltham, MA, USA). The perovskite solar cell was assembled using the modified method using a triple-cation type lead halide perovskite that has been demonstrated previously [14].

After the sintering process of ETL, UV–ozone treatment was conducted prior to spin-coating the halide perovskite precursor solution. Nitrogen was flowed into a glove box to keep the humidity at around 20 %. The substrates were placed in the glove box and heated to 80 °C on a hot plate. To prepare a perovskite precursor solution with a composition of Cs_0.05_MA_0.1_FA_0.85_PbI_2.9_Br_0.1_·0.05PbI_2_, MABr 0.14 mmol (0.0157 g), CsI 0.07 mmol (0.0182 g), FAI 1.19 mmol (0.204 g), and PbI_2_ 1.45 mmol (0.666 g) were dissolved in the mixed solvent of DMF 80 vol% (0.8 mL) and DMSO 20 vol% (0.2 mL). It was then stirred on a hot plate in the dark at 80 °C for 2 h. The heated substrates at 80 °C were set on the spin-coater, and 100 μL of perovskite precursor solution was dropped onto the substrate. A two-step program of spin-coater (1000 rpm for 10 s and 6000 rpm for 30 s) was used. A 200 μL volume of CB was dropped with an electronic micropipette 25 s after the start of spin-coating. The substrate was annealed using a hot plate at 100 °C for 1 h, and it was allowed to cool naturally to room temperature. Then, 0.12 mmol (0.0148 g) of FABr was added to 4 mL of IPA to prepare a solution (30 mM), and it was spin-coated on top of the crystallized perovskite layer. A 100 μL volume of the prepared FABr solution was coated at 3000 rpm for 30 s. The substrate was heated on a hot plate at 80 °C for 10 min. After heating, the substrate was allowed to cool to room temperature.

To prepare a solution for the hole transporting layer (HTL), 0.037 mmol (0.045 g) of Spiro-MeOTAD was dissolved in 0.5 mL of CB. Next, 0.18 mmol (0.052 g) of Li-TFSI was dissolved in 0.1 mL of acetonitrile. A 10 μL volume of the acetonitrile solution and 17.75 μL of TBP were added to the solution containing Spiro-MeOTAD. The substrate was cooled to room temperature and spin-coated at 3000 rpm for 30 s. After 5 s from the start of spin-coating, 60 μL of the solution was dropped. The substrates were left for 24 h. Finally, a gold layer was deposited on top of Spiro-MeOTAD by using the thermal evaporation method [15].

Current–voltage curves were obtained under AM 1.5 simulated sunlight (100 mW/cm^2^) using a solar simulator (Yamashita Denso, YSS-80A, Tokyo, Japan) and potentiostat (Hokuto Denko HSV-110, Tokyo, Japan). The active area of the device was 0.09 cm^2^. The current–voltage curves were measured under reverse scanning conditions at a scan rate of 50 mV/s.

## 3. Results and Discussions

The fluid containing precursor materials of tin and titanium ions plays a vital role in determining the resultant film characteristics, such as uniformity of the obtained nanoparticle layers and density of nanocrystals in the film. We attempted to establish a sintering protocol of Sn/Ti oxide thin films applicable for electron transport layers in PSCs. In principle, base liquids and/or additive compounds in the fluid are required to optimize both the concentration of the metal ion sources and the packing structure of formed nanoparticles during heat treatment. By increasing the temperature, hydrolysis and polycondensation of those metal ions start to promote the oxide nanoparticle formation, where the homogeneous reactions should be adjusted in the precursor film. In addition to the use of both ethanol and terpineol to dissolve metal ions, we added a PEG polymer in order to disperse the obtained nanoparticles in the film. PEG is well-known to stabilize metal oxide nanoparticles preventing agglomeration, such as TiO_2_. The sizes of those nanoparticles can be reduced by steric repulsion.

The preliminary experiments allowed us to use the mixed base liquid, i.e., ethanol/terpineol, because the resultant fluid using only ethanol did not give a uniform film after the sintering treatment and instead some cloudy spots were observed in the film. We anticipated that terpineol would be a key chemical, as has been disclosed in the nanoparticle sintering research on dye-sensitized solar cells [16]. In this device, TiO_2_ nanoparticle films, which is the host material of light-absorbing dye molecules, can be prepared by a similar sintering approach with a precursor solution comprising suitable base liquids and additives.

**Figure 2:**
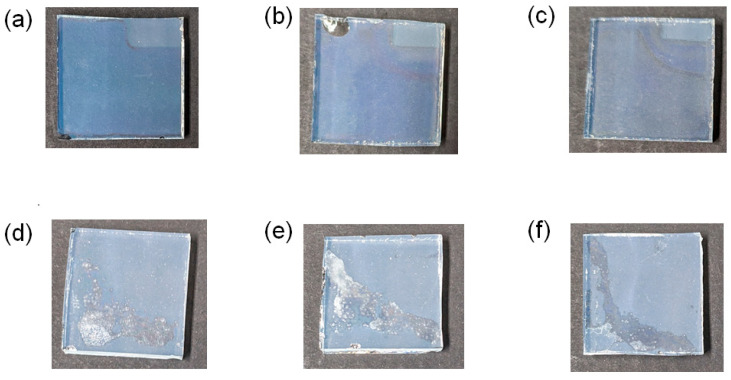
Photos of several Sn/Ti oxides-deposited FTO glass substrates after heat treatment at 500 °C. Samples (**a**–**c**) were prepared by using a precursor solution of tin and titanium compounds dissolved in a mixed ethanol/terpineol base liquid. The ratios of tin and titanium ions (Sn/Ti) in the precursor were 25:75 for (**a**), 50:50 for (**b**), and 100:0 for (**c**), respectively. Samples (**d**–**f**) were prepared via the same sintering strategy as those of (**a**–**c**). An ethanol base liquid without terpineol was employed for the preparation of samples (**d**–**f**). Similarly, the ratios of Sn/Ti in the precursor were 25:75 for (**d**), 50:50 for (**e**), and 100:0 for (**f**), respectively. For samples (**a**–**c**), the right parts highlighted in a dotted yellow line were not covered with Sn/Ti oxide thin films for fabricating perovskite solar cells. The three samples (**d**–**f**) showed the formation of agglomerated Sn/Ti oxides on top of the FTO substrates, whereas uniform films formed for (**a**–**c**) through the optimization of the composition of precursor solutions.

The concentration dependence of tin and titanium ions on the film quality was systematically examined, and typical photos of the processed films after sintering are depicted in Figure 2. White agglomerated particles formed when terpineol was not used as a base liquid (Figure 2d–f), whereas transparent compact thin films with an approximate thickness of 100 nm as mentioned later were obtained for the ethanol/terpineol base liquid (Figure 2a–c). It appears that the terpineol with the higher boiling point (~100 °C) than ethanol assisted the homogenous reaction as a solvent, leading to the formation of well-dispersed nanoparticle films. It is likely that PEG also worked as a base liquid similar to the other solvents in a way that the inhomogeneous reactions were retarded due to the higher boiling temperature (>200 °C) than those of ethanol and terpineol.

**Figure 3:**
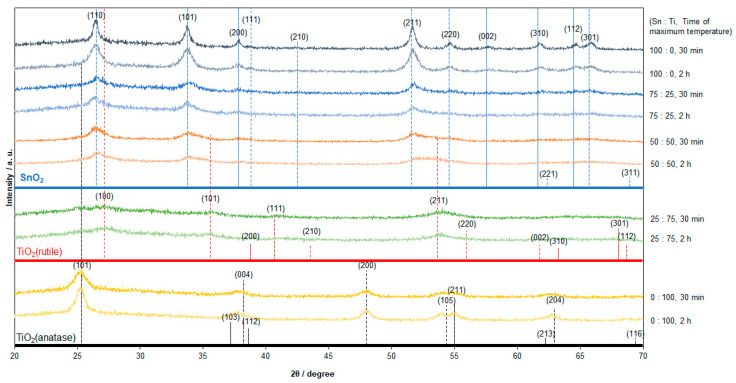
XRD pattern of the sintered Sn/Ti oxide samples at 500 °C in air. The ratios of Sn/Ti in the precursor were 100:0, 75:25, 50:50, 25:75, and 0:100. The database of SnO_2_ (blue, JCPDS: 01-077-0452), rutile TiO_2_ (red, JCPDS: 01-076-0324), and anatase TiO_2_ (blue, JCPDS: 01-075-2550) is also presented. The period of maximum temperature in the sintering was 30 min and 2 h.

X-ray diffraction patterns (XRD) of the Sn/Ti oxide samples sintered at 500 °C were analyzed to structurally identify crystalline compounds. Figure 1 depicts the two sintering strategies changing the ramping time, (a) 2 h and (b) 30 min, and periods of maximum temperature, (a) 2 h and (b) 30 min. As a result of heat treatment, XRD patterns exhibiting several broad peaks were obtained, which are presented in Figure 3.

The observed peaks for the measurements (Sn/Ti = 100:0, 75:25, and 50:50) were basically assigned to be those of rutile-phase SnO_2_. As indicated by the database of SnO_2_ (JCPDS:01-077-0452) and TiO_2_ (JCPDS: 01-076-0324), those peaks with similar diffraction angles, such as (110) peaks, hindered detailed analysis on crystallite sizes. Using the peaks corresponding to the (101) plane of SnO_2_, crystallite sizes were estimated from the Scherrer equation (1). D, K, λ, and θ indicate the crystallite size of SnO_2_, Scherrer constant (= 0.90), X-ray wavelength (= 1.54 Å), and Bragg angle, respectively.

D = Kλ/βcosθ(1)

For 30 min sintering samples, the estimated crystallite sizes were 19 nm, 3 nm, and 6 nm for 100:0, 75:25, and 50:50, respectively. For 2 h sintering, the crystallite sizes were 4 nm, 3 nm, and 3 nm for 100:0, 75:25, and 50:50, respectively. Additionally, it turned out that TiO_2_ was not well crystallized for all those conditions. The more efficient crystallization at lower temperatures for SnO_2_ probably suppressed the TiO_2_ crystallization during the heat treatment. The time-dependent analysis via heat treatment suggested that crystal growth was not enhanced with the prolonged heating.

In contrast, TiO_2_ crystallization was observed both for Sn/Ti = 25:75 and 0:100. For the smaller amount of Sn source (Sn/Ti = 25:75), it is likely that one of the components of the base liquid, i.e., PEG, retards the crystal growth of SnO_2_, allowing to produce the Rutile TiO_2_ with estimated crystal sizes of less than 2 nm. Since SnO_2_ has a tendency to form a rutile crystal face, this may lead to the Rutile TiO_2_ formation, whereas for 0:100, only Anatase TiO_2_ grew as a result of the heat treatment.

**Figure 4:**
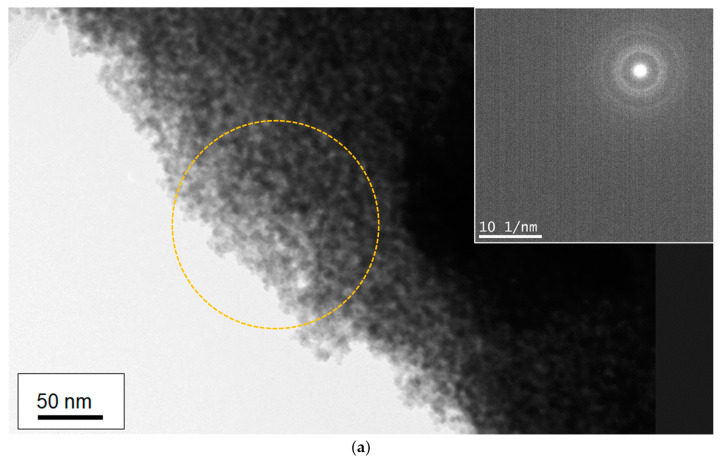

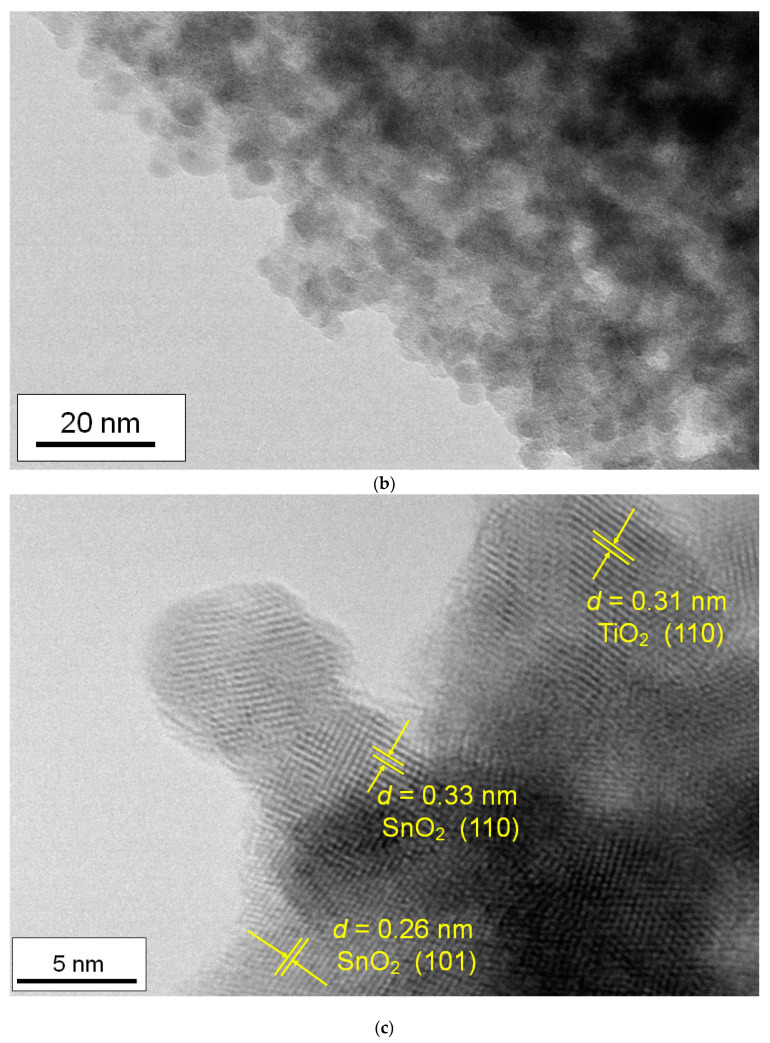

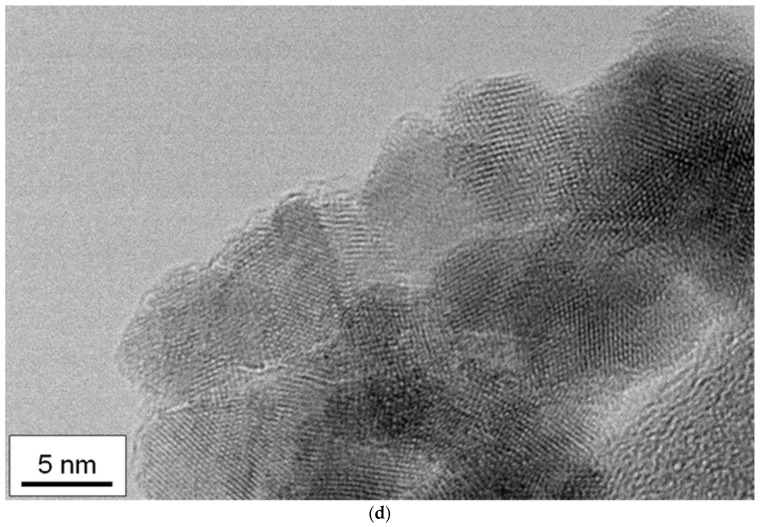
TEM images of the Sn/Ti oxide sample treated at 500 °C in air. The ratio of Sn/Ti in the precursor solution was 50:50. The period of maximum temperature in the sintering was 2 h. (**a**,**b**) Lower-resolution images of the Sn/Ti oxide sample. Inserted image in (**a**) presents the Selected Area Diffraction (SAD) pattern corresponding to the observed area of interest highlighted with a dotted orange circle. (**c**,**d**) High-resolution TEM images (HR-TEM) measured at two different positions. Figure (**c**) shows a *d*-spacing for the observed lattice fringes and lattice planes corresponding to (110) and (101) of SnO_2,_ and (110) of TiO_2_.

**Figure 5:**
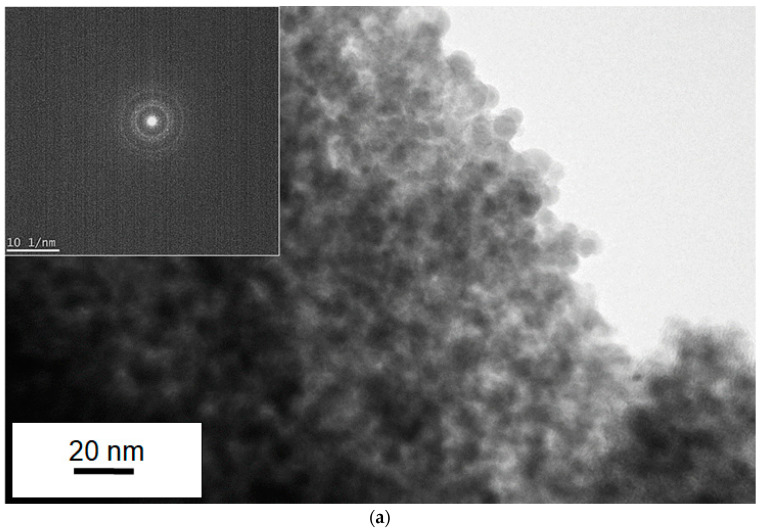

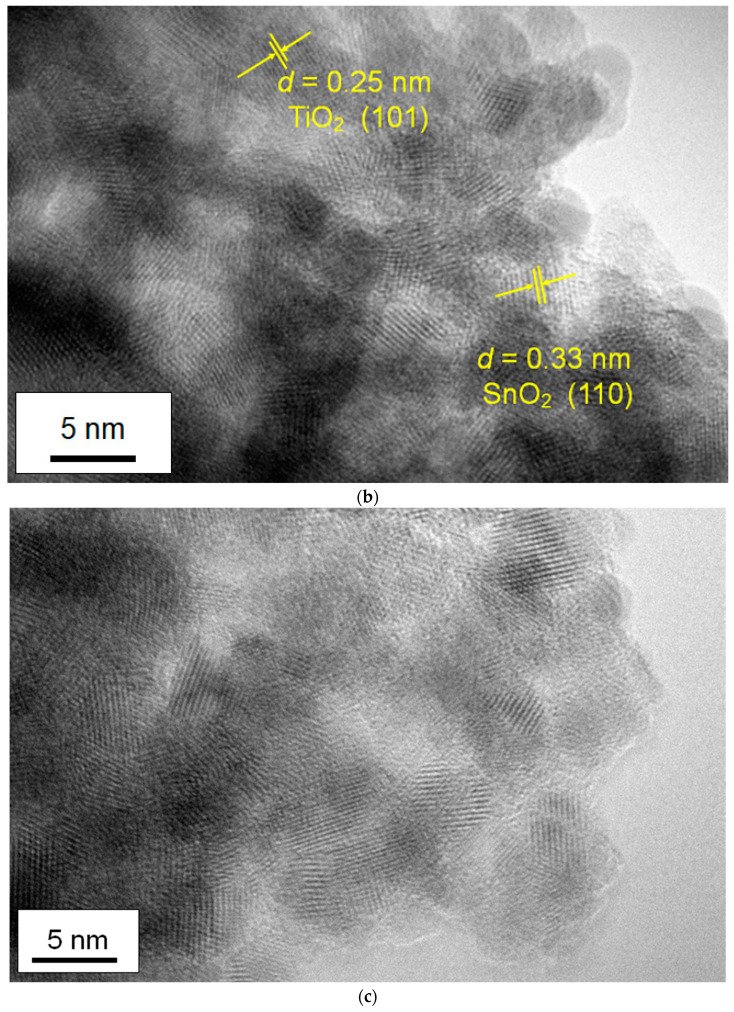
TEM images of the Sn/Ti oxide sample treated at 500 °C in air. The ratio of Sn/Ti in the precursor solution was 50:50. The period of maximum temperature in the sintering was 30 min. (**a**) Lower-resolution image of the Sn/Ti oxide sample. Inserted image in (**a**) presents the SAD pattern. (**b**,**c**) HR-TEM images measured at two different positions. (**b**) shows a *d*-spacing for the observed lattice fringes and lattice planes corresponding to (110) of SnO_2_ and (101) of TiO_2_.

To investigate the mechanism of film formation of Sn/Ti oxide hybrid films, a transmission electron microscope (TEM) was utilized in order to clarify the microstructure of nanoparticles at a single nanometer scale associated with the formation of mixed oxide layers from titanium and tin compounds. Figure 4 and Figure 5 present several TEM images obtained with different resolutions (Sn/Ti = 50:50), where the images from different positions are also depicted to show the observed uniformity of the samples. The samples prepared by two different sintering strategies (as shown in Figure 1) were measured, and the results are separately illustrated in Figure 4 and Figure 5. As can be seen in Figure 4a,b, it turned out that single-nanometer-scale particles with the approximate dimension of 3~5 nm were formed during heat treatment. The HR-TEM images in Figure 4c indicate lattice fringes exhibiting the (110) and (101) planes of rutile crystal-phase SnO_2_ and the (110) plane of rutile TiO_2_. These d-spacings were 0.33 nm, 0.26 nm, and 0.31 nm, respectively. In fact, the calculated crystallite size was consistent with that of the primary nanocrystal size observed in the images that were measured at different positions (Figure 4d). Irregular-shaped nanocrystals as a result of their fusion were more striking for the longer sintering (the period of maximum temperature: 2 h) than those of 30 min sintering (Figure 5c,d). In other words, more densely packed films were obtained for the 2 h sintering. Similarly, it was found from HR-TEM images of Figure 5c that lattice fringes were assigned to the (110) of rutile SnO_2_ and the (101) plane of rutile TiO_2_. These d-spacings were 0.33 nm and 0.25 nm, respectively.

**Figure 6:**
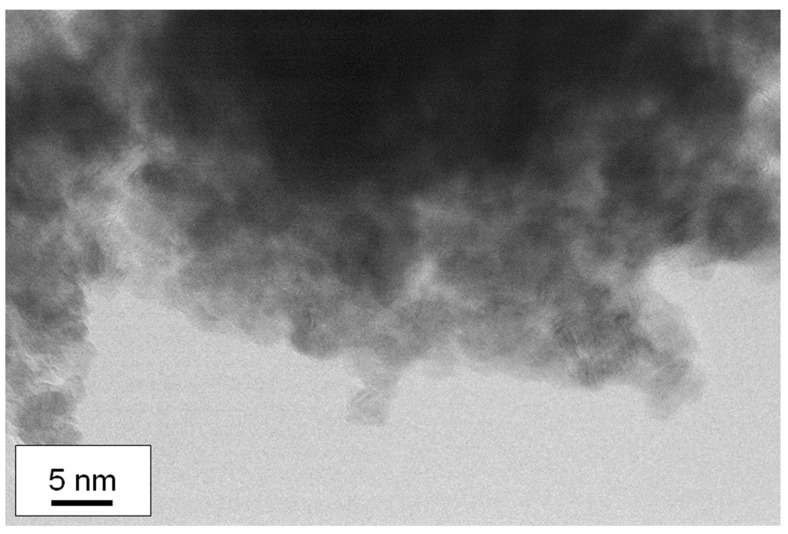
A TEM image of the Sn/Ti oxide sample treated at 500 °C in air (the ratio of Sn/Ti in the precursor solution was 25:75). The period of maximum temperature in the sintering was 30 min.

In contrast, in the Sn/Ti oxide hybrid film prepared with Sn/Ti = 25:75 condition (Figure 6), the TEM measurement showed very weak crystallinity. This made it difficult to estimate the primary particle sizes because of the aggregation of the particles.

**Figure 7:**
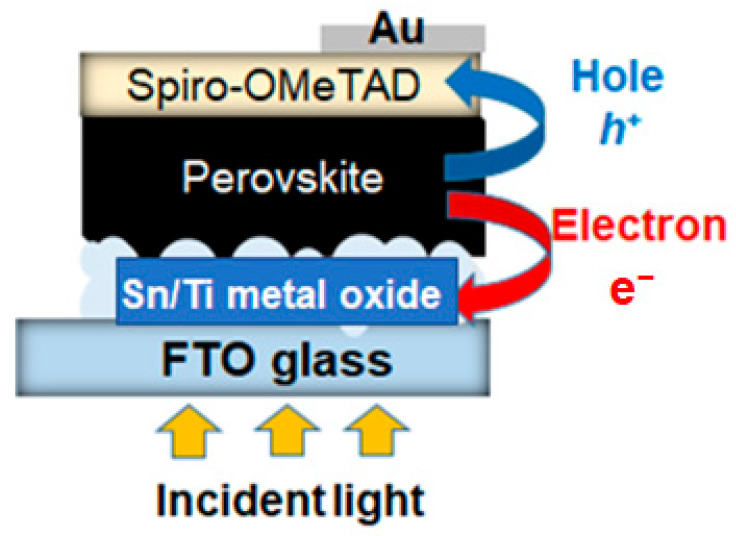
A device structure of the lead halide PSCs tested in this work. The ETL consists of a Sn/Ti oxide thin film (single layer) deposited on top of the FTO glass.

Figure 7 illustrates a device structure of PSCs employing Sn/Ti oxide films as the electron transport layer (ETL) evaluated in this work. In principle, the light absorption of the perovskite layer sandwiched between an ETL and the hole transporting layer (HTL) enables charge separation in PSCs. The photocarriers (e^−^ and h^+^) can be moved to each electrode to achieve photoenergy conversion. Here, our focus was centered on the ratio of Sn/Ti in the precursor solutions of terpineol/PEG base liquids that can optimize the light-to-electricity conversion efficiency of the device.

**Figure 8:**
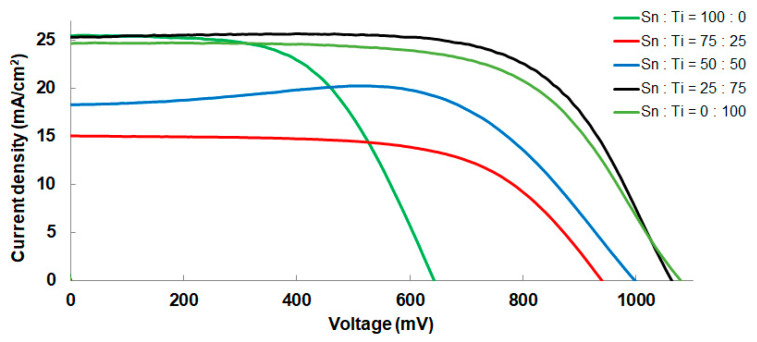
I–V curves of the best-performing devices by Sn/Ti oxide ETLs measured under simulated sunlight. The period of maximum temperature in the sintering of ETL was 30 min.

**Figure 9:**
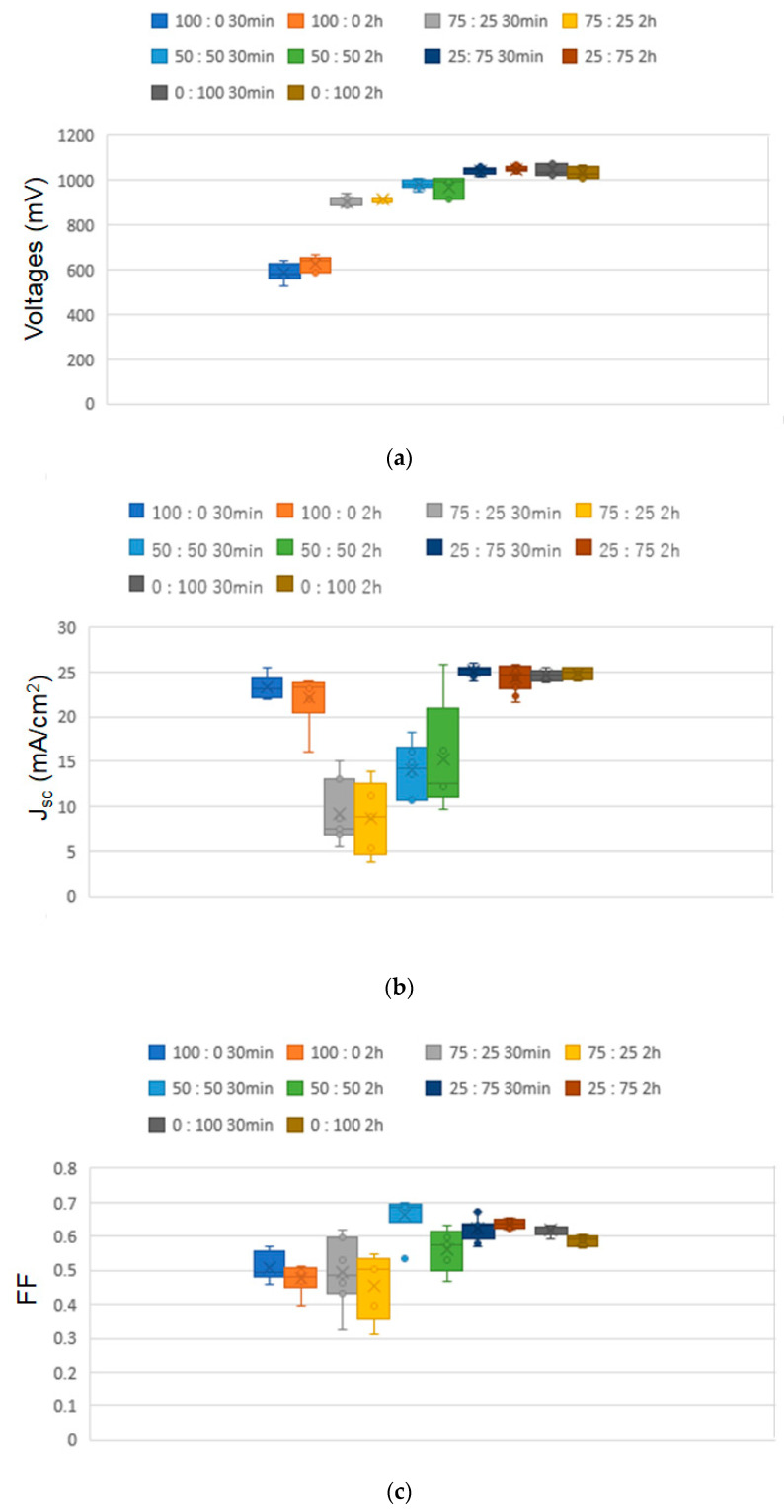

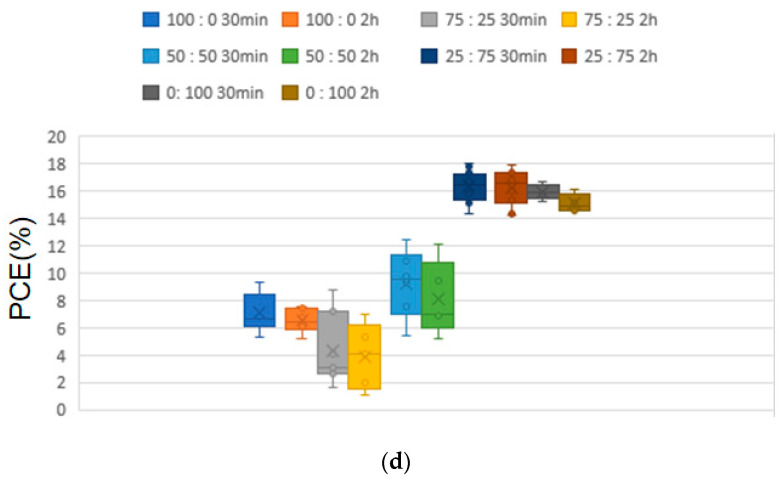
Distributions of I–V parameters of (**a**) V_oc_, (**b**) J_sc_, (**c**) FF, and (**d**) PCE. All the data were obtained from each solar cell fabricated using the 10 different ETLs.

Figure 8 shows the I–V curve of the best-performing cell measured under one sunlight irradiation (100 mW/cm^2^). Additionally, Table 1 summarizes the I–V parameters. A maximum power conversion efficiency (PCE) of 18.1% was obtained for the solar cell fabricated using a sintering strategy (ramping time: 30 min, period of maximum temperature: 30 min) when the Sn/Ti was 25:75. Figure 9 presents the distributions of I–V parameters obtained from each solar cell consisting of the 10 different ETLs. The data clearly show that there is a correlation between J_sc_ and PCE. In other words, the enhanced J_sc_ led to a marked improvement in PCEs. Considering that the samples (Sn/Ti = 100:0, 75:25, 50:50) had higher crystallinity compared to those of Sn/Ti = 25:75, it is most likely that the more effective electron transport took place for the 25:75 film-based solar cells with low-crystallinity ETLs. In this regard, Dorman et al. reported the increased charge mobility of high-temperature processed Sn-doped TiO_2_, whereas charge carrier recombination can be decreased in solar cells [11]. Since the ionic radius of Sn(IV) is 69 p.m., which is larger than Ti(IV) (61 p.m.) [17], it is thought that Sn-doped TiO_2_ lowered the crystallinity, and the ETL had positive effects on the improved PCEs.

**Figure 10:**
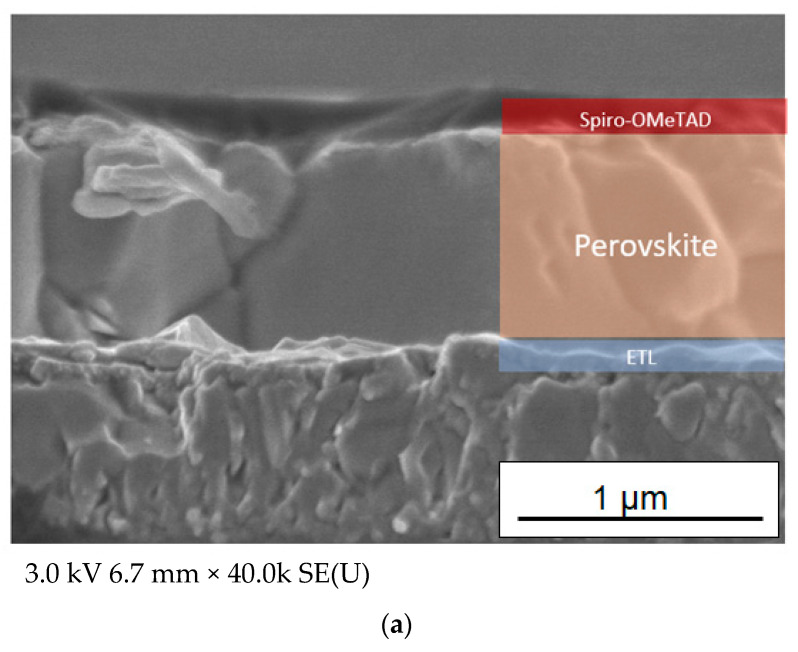

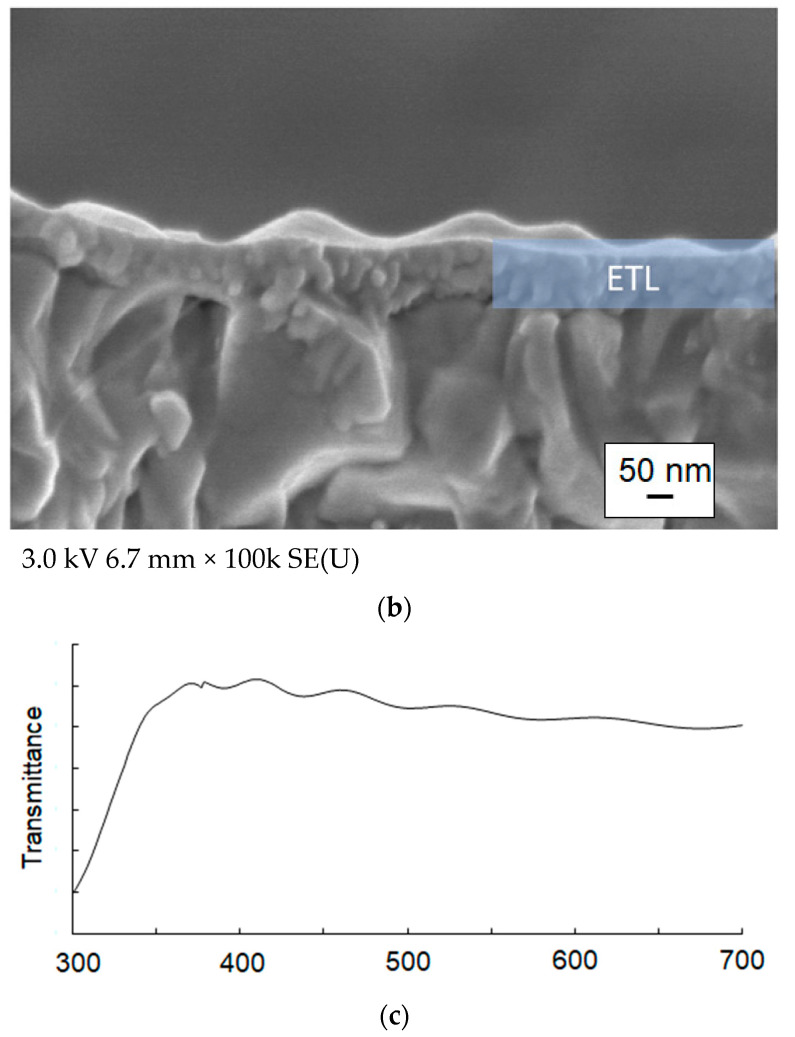
Cross-sectional SEM images of the best-performing device: (**a**) low-magnification image and (**b**) high-magnification image presenting the ETL part; (**c**) transmittance spectrum of the ETL thin film, which is the same sample as (**b**).

As shown in Figure 10a, the lead halide perovskite had a grain boundary even for the best-performing device. The approximate thickness of the ETL was 100 nm (Figure 10b). The cross-sectional SEM image in Figure 10b enabled us to observe the smooth surface of the thin film. In addition, the transmittance spectrum of the Sn/Ti oxide film in Figure 10c showed that the incident light can ensure efficient absorption in the perovskite layer in the device. The optimization of the deposition condition of the perovskite precursor solution and ETL thickness will further improve efficiencies by the smaller grain boundary formation of perovskite and the efficient electron generation and transport at the material interface. Most notably, a simple one-time deposition process of the Sn/Ti ETL using the mixed ethanol/terpineol/PEG base liquid made it possible to realize the respectable PCE.

## 4. Conclusions

Nanoparticle-based uniform Sn/Ti metal oxide films were successfully obtained via sintering through optimization of the composition of precursor solutions containing titanium and tin salts, which were dissolved in an ethanol/terpineol/PEG base liquid. It was suggested that PEG also had a key role in improving the homogeneity of the film during sintering.

We demonstrated that the maximum power conversion efficiency was obtained for the concentration condition of [SnCl_2_·2H_2_O]/[titanium tetraisopropoxide (TTIP)] = 25:75 in the terpineol/PEG-based precursor solution. The combination of XRD and high-resolution TEM analysis was a straightforward approach to understanding the microstructures related to the crystallinity and fusion of nanoparticles depending on the sintering strategies. It appears that the lower crystallinity for the 25:75 Sn/Ti oxide films has significant potential that boosts the power conversion efficiency of the device. Such fundamental information on semiconductor Sn/Ti oxide hybrid layers will be beneficial for the further development of cost-effective high-performance perovskite solar cells [18].

## Figures and Tables

**Table 1:** I–V parameters obtained for the best-performing perovskite solar cell fabricated using the 10 ETLs with different ratios of Sn/Ti and sintering times.

Sn:Ti	Time	V_oc_ (mV)	J_sc_ (mA/cm^2^)	FF	PCE (%)
**100:0**	**30 min**	643	25.47	0.571	9.3
	**2 h**	646	23.12	0.508	7.6
**75:25**	**30 min**	939	15.04	0.620	8.8
	**2h**	922	13.90	0.548	7.0
**50:50**	**30min**	997	18.29	0.686	12.5
	**2 h**	1006	25.77	0.469	12.2
**25:75**	**30 min**	1063	25.32	0.671	18.1
	**2 h**	1057	25.90	0.657	18.0
**0:100**	**30 min**	1078	24.66	0.628	16.7
	**2 h**	1068	25.10	0.601	16.1

## Data Availability

Not applicable.
